# Association of Left Ventricular Function With Cerebral Small Vessel Disease in a Community‐Based Population

**DOI:** 10.1111/cns.70226

**Published:** 2025-02-20

**Authors:** Yingying Yang, Xueli Cai, Mengyuan Zhou, Yiyi Chen, Jingtao Pi, Mengxi Zhao, Yulu Shi, Jing Jing, Weiqi Chen, Hongyi Yan, Xia Meng, Yongjun Wang, Yuesong Pan, Yilong Wang

**Affiliations:** ^1^ Department of Neurology, Beijing Tiantan Hospital Capital Medical University Beijing China; ^2^ Department of Neurology The Fifth Affiliated Hospital of Wenzhou Medical University Lishui China; ^3^ China National Clinical Research Center for Neurological Diseases Beijing China; ^4^ Chinese Institute for Brain Research Beijing China; ^5^ National Center for Neurological Disorders Beijing China; ^6^ Advanced Innovation Center for Human Brain Protection Capital Medical University Beijing China; ^7^ Beijing Laboratory of Oral Health Capital Medical University Beijing China; ^8^ Beijing Municipal Key Laboratory of Clinical Epidemiology Beijing China; ^9^ Laboratory for Clinical Medicine Capital Medical University Beijing China

**Keywords:** cerebral small vessel disease, community‐based cohort, diastolic function, left ventricular function

## Abstract

**Background and Objectives:**

The relationship of cardiac function with cerebral small vessel disease (CSVD) remains unknown. The study aimed to investigate the association between left ventricular (LV) function and CSVD in a community‐based population.

**Methods:**

Community‐dwelling residents in China from the cross sectional survey of the PRECISE (PolyvasculaR Evaluation for Cognitive Impairment and vaScular Events) cohort were included. LV ejection fraction (LVEF) and LV fractional shortening (LVFS) were measured for LV systolic function, and mitral E/A ratio (the ratio of the peak trans‐mitral filling velocity during early diastole and late diastole) was evaluated for LV diastolic function by transthoracic echocardiogram (TTE). Total CSVD score and CSVD imaging makers including white matter hyperintensity (WMH), lacunes, cerebral microbleeds (CMB), and enlarged perivascular spaces (EPVS) were assessed. The associations of cardiac function with CSVD were analyzed using ordinal or binary logistic regression models. Restricted cubic spline models fitted for logistic regression models were used.

**Results:**

A total of 3063 participants with available TTE and brain MRI data were included in the study. In the multivariable logistic regression analysis, LVEF and LVFS were not associated with total CSVD score or markers of CSVD. E/A ratio showed a negative correlation with total CSVD score (cOR, 0.89, 95% CI: 0.80–0.98, *p* = 0.01). Participants with E/A ≤ 0.8 or ≥ 2 had a higher total CSVD score than those with 0.8 < E/A < 2 (cOR 1.20, 95% CI: 1.00–1.43, *p* = 0.046). E/A ratio was also correlated with lacunes, moderate to severe EPVS, and periventricular WMH. Logistic regression analyses with restricted cubic spline further demonstrated that a lower E/A ratio were associated with a higher total CSVD score.

**Conclusion:**

Our study showed that mitral E/A ratio was associated with nonhemorrhagic CSVD. LV diastolic dysfunction assessed by TTE provides clues for the early warning of high CSVD burden.

## Graphical abstract

Our study showed that mitral E/A ratio was associated with nonhemorrhagic cerebral small vessel disease (CSVD). Left ventricular diastolic dysfunction assessed by transthoracic echocardiogram provides clues for the early warning of high burden of CSVD.

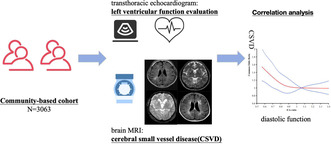

## 1 Introduction

Cerebral small vessel disease (CSVD) is a disorder of the brain's small perforating arterioles, capillaries and venules, causing major health problems including ischemic stroke, vascular dementia and intracerebral hemorrhage [1]. Lacune, white matter hyperintensity (WMH), enlarged perivascular spaces (EPVS) and cerebral microbleed (CMB) are common and important markers of CSVD on brain magnetic resonance imaging (MRI) [2, 3]. The pathogenesis of CSVD is poorly understood but there is evidence of a role for chronic cerebral hypoperfusion [4, 5]. The heart plays a role in maintaining adequate perfusion of the brain and they also share similar risk factors. WMH is frequently noted in patients with chronic cardiac disease, which indicates long‐term alteration of cardiac functions, and cardiac function might have an influence on the development of WMH [6, 7, 8]. The evaluation of cardiac function in association with CSVD may help us to elucidate the pathophysiological role of the heart in the development of CSVD.

Left ventricular ejection fraction (LVEF) is a marker of cardiac systolic function. Cardiac systolic function was associated with CSVD in acute ischemic stroke patients [9], but studies on its association with CSVD in the general population were inconsistent. A cross sectional analysis of UK Biobank showed that LVEF had a negative linear association with WMH [10]. In a community‐based cohort without overt cardiac disease, instead of LVEF, lower global longitudinal strain (GLS) which can detect subclinical systolic abnormalities even when LVEF is normal was shown to be independently associated with silent brain infarcts and WMH [11]. Meantime, the relationship of cardiac diastolic function with CSVD in community‐based population remains unclear. A previous study indicated that cardiac diastolic function might be associated with WMH [12]. Transthoracic echocardiogram (TTE) is the primary noninvasive modality for the assessment of cardiac function. The aim of the present study is to investigate the association between cardiac function assessed by TTE and CSVD in a community‐based cohort. We hypothesized that cardiac function might be associated with CSVD.

## 2 Materials and Methods

### 2.1 Study Participants

The participants were from the PolyvasculaR Evaluation for Cognitive Impairment and vaScular Events (PRECISE) study. Details of the rationale and design of the PRECISE study have been described previously [13]. Mainly, the PRECISE study is a population‐ based prospective cohort aiming to establish the prevalence of clinical or subclinical polyvascular lesions using advanced vascular imaging techniques. Between May 2017 and September 2019, 3067 community‐dwelling residents aged 50–75 years from six villages and four communities of Lishui city in southeastern China were eligible and enrolled at baseline. Exclusion criteria of this study included participants with contraindications to MRI and computed tomography angiography (CTA), life expectancy ≤ 4 years and mental diseases. This study was approved by the ethics committees of both Beijing Tiantan Hospital (IRB approval number: KY2017‐010‐01) and Lishui Hospital (IRB approval number: 2016‐42). Informed consent was obtained from all subjects enrolled in the PRECISE study.

### 2.2 Clinical Assessment

All the participants underwent questionnaire assessments, clinical examinations and laboratory tests at the Lishui hospital through face‐to‐face interviews and examinations by centralized trained personnel. We assessed demographic, clinical, and cardiovascular risk factors, including age, sex, smoking status, mean systolic blood pressure (SBP), mean diastolic blood pressure (DBP), medical history (hypertension, diabetes, hyperlipidemia, stroke, and cardiac disease), education level, total cholesterol (TC), low density lipoprotein cholesterol (LDL‐C), fast blood glucose (FBG), and homocysteine (HCY). Venous blood samples routinely drawn after an overnight fast were analyzed for plasma TC, LDL‐C, FBG, and HCY.

### 2.3 The Assessment of Cardiac Function

Comprehensive 2‐dimensional and Doppler TTE examinations were performed using a commercially available system with 2.5‐MHz transducers by skilled echocardiographers following a standardized protocol [14]. The TTE data were collected blinded to brain MRI information. To measure LV systolic function, LVEF was obtained using the Simpson's method from apical 2‐ and 4‐ chamber views and LVEF > 50% usually indicates normal systolic function. Left ventricular fractional shortening (LVFS) was collected as another systolic function marker. To evaluate LV diastolic function, we used the following variables [15]. The peak trans‐mitral filling velocity during early diastole (E), late diastole (A) and deceleration time (DT) of mitral E velocity were imaged at the tip of the mitral leaflets from an apical 4‐chamber view. E velocity is dependent on left atrial (LA)‐LV pressure gradient in early diastole and therefore LV relaxation and LA pressure. A velocity depends on LA‐LV pressure gradient during late diastole, and therefore LV stiffness and LA contractility. E/A ratio ≤ 0.8 or E/A ratio ≥ 2 usually suggest LV diastolic dysfunction [15]. DT is influenced by the rate of decline in LA‐LV pressure gradient after mitral valve opening and therefore LV stiffness. When LV relaxation is slow, LV relaxation also affects DT. Color‐coded tissue Doppler imaging was applied to the apical 4 chamber view to determine mean early velocity at the septal mitral annulus (e′). e′ velocity is dependent on LV relaxation. As e′ velocity corrects for the effect of LV relaxation on E velocity, E/e′ ratio > 14 indicates LV filling pressure. Structural parameters from TTE including left atrial diameter (LAD) and left ventricular end‐diastolic diameter (LVEDD) were also measured.

### 2.4 Imaging Analysis of CSVD

Brain MRI scans were performed on a 3.0 T scanner (Ingenia 3.0 T, Philips, Best, The Netherlands) at baseline. Both TTE and brain MRI scans were done within 7 days after enrollment of participants. Sequences included T1, T2, fluid‐attenuated inversion recovery and susceptibility‐ weighted imaging. The detailed scanner parameters were listed in Table S1. Imaging data were collected in digital imaging and communications in medicine format on discs and analyzed by the imaging research center at Beijing Tiantan Hospital.

Four markers of CSVD were analyzed following the STandards for ReportIng Vascular changes on nEuroimaging criteria, including lacunes, WMH, CMB, EPVS [2, 3]. Periventricular WMH (PWMH) and deep WMH (DWMH) were graded separately on FLAIR according to the Fazekas scale [16]. We defined PWMH of Fazekas scale = 3 or DWMH of Fazekas scale ≥ 2 as confluent WMH [17]. Anatomic localization of CMB was classified into three categories: lobar (cortical gray and subcortical white matter), deep (deep gray matter: basal ganglia and thalamus, white matter of the corpus callosum, and internal, external, and extreme capsules), and infratentorial (brainstem and cerebellum). The number of CMBs were recorded in different areas. We rated EPVS on T2 imaging in the basal ganglia as grades 0 = none, 1 = 1–10, 2 = 11–20, 3 = 21–40, and 4 > 40 per side using the worse side if there was any asymmetry. We defined EPVS in the basal ganglia of grades 2–4 as moderate to severe EPVS. The total CSVD score was rated on an ordinal scale from 0 to 4, by counting the presence of four MRI features of CSVD [18]. A point was awarded for each of the following: presence of lacune, confluent WMH, moderate–severe EPVS, and presence of CMB.

Imaging assessment of each CSVD marker was rated by two raters who were blinded to patients' clinical data. Images with inconsistent results were finally assessed by another senior neurologist who were blinded to initial results. The kappa coefficient of CSVD markers on brain MRI between raters were as follows: 0.80 for the presence of lacune, 0.82 for Fazekas scale of WMH, 0.80 for the presence of CMB, and 0.90 for the severity of EPVS. Details of imaging analysis of CSVD have been described previously [19, 20, 21].

### 2.5 Statistical Analyses

Categorical variables are presented as frequencies and percentages, whereas continuous variables are presented as mean with standard deviation (SD) or median with interquartile range (IQR). The baseline characteristics were compared between participants with high CSVD burden (total CSVD scores = 2–4) and low CSVD burden (total CSVD scores = 0–1). Kolmogorov–Smirnov test for normality were used to assess data distribution. Categorical variables were compared with Chi‐square test or Fisher's exact test, whereas continuous variables were compared with Student's *t*‐test and Wilcoxon test.

The association of TTE parameters related to cardiac function (per SD) with CSVD were evaluated. For total CSVD score, ordinal logistic regression models were conducted and common odds ratios (cOR) with their 95% confidence intervals (CI) were calculated. For CSVD imaging markers, binary logistic regression models were performed and odds ratios (OR) with their 95% CI were presented. Potential covariates were chosen according to previous literature and were adjusted in multivariable logistic regression models, including age, sex, current smoker, SBP, DBP, education level, history of hypertension, history of diabetes, history of hyperlipidemia, history of stroke, TC, LDL‐C, FBG and HCY. Participants were further divided into 2 groups according to E/A ratio (E/A ≤ 0.8 or ≥ 2 and 0.8 < E/A < 2). Taking the normal range (0.8 < E/A < 2) as the reference, multivariable logistic regression models were performed to explore the correlation between E/A ratio and total CSVD score or CSVD markers. We performed ordinal logistic regression models with restricted cubic splines for E/A ratio (continuous variable) with adjustments for all potential covariates to the pattern and magnitude of associations between E/A ratio and total CSVD score. The 5th, 25th, 50th, 75th, and 95th percentiles of the E/A ratio were used as the 5 knots for spline.

Two‐sided *p* < 0.05 were considered statistically significant. All analyses were performed using SAS software version 9.4 (SAS Institute Inc., Cary, North Carolina).

### 2.6 Patient and Public Involvement

The study design, analysis, interpretation of data and drafting of the manuscript did not involve any patients or members of the public.

## 3 Results

### 3.1 Baseline Characteristics of Participants

**FIGURE 1:**
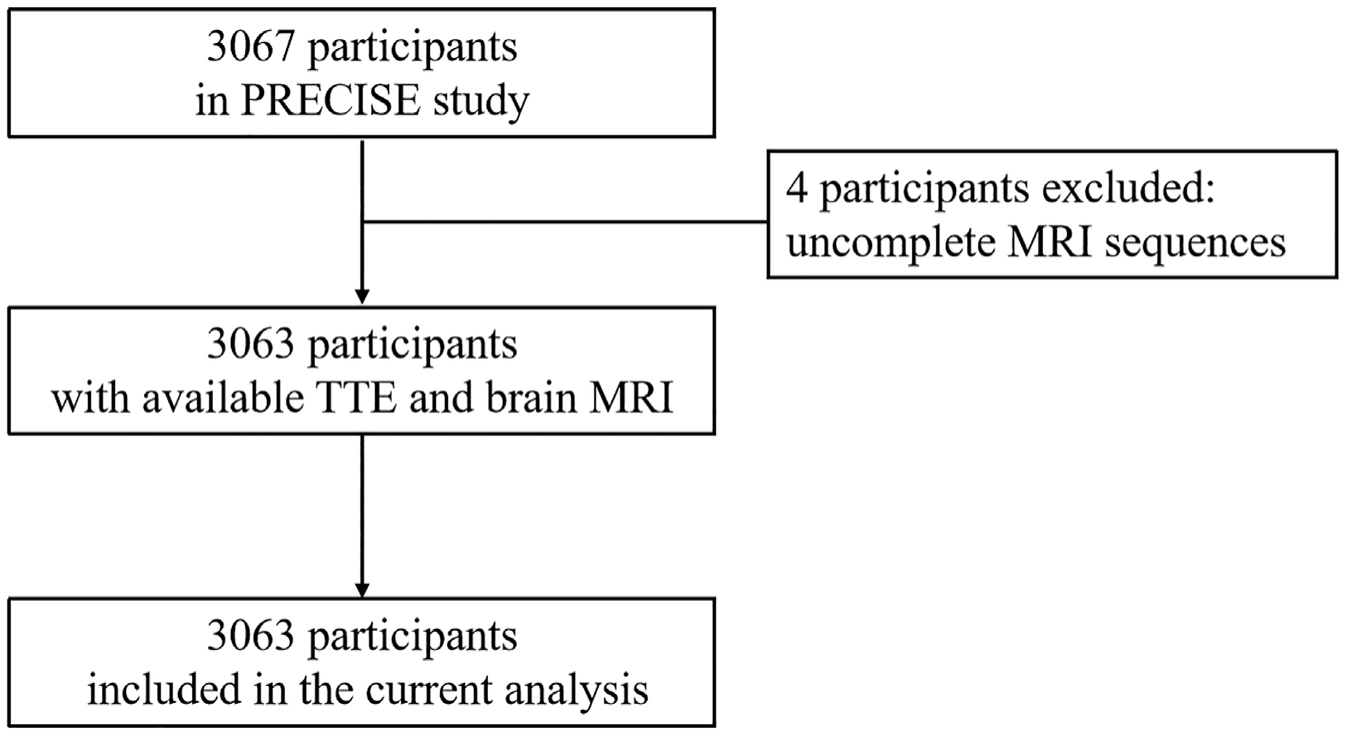
Flowchart of participant selection in the study. TTE, transthoracic echocardiogram.

Among 3067 participants enrolled in PRECISE study, four participants were excluded because of motion artifacts or missing MRI sequences. Therefore, 3063 participants with available TTE and MRI data were included in this analysis (Figure 1). The mean age of all the participants were 61.2 ± 6.7 years, and 46.5% of them were male. The prevalence of lacune, confluent WMH, moderate–severe EPVS, and CMB were 5.6%, 16.7%, 9.8%, and 10.2%, respectively. The total CSVD scores were 0, 1, 2, 3, and 4 points in 2129, 679, 176, 54, and 25 participants, respectively. Comparison of baseline characteristics in participants with high CSVD burden (total CSVD scores = 2–4, *n* = 255) and low CSVD burden (total CSVD scores = 0–1, *n* = 2808) were displayed in Table 1. Participants with high CSVD burden had a higher proportion of elderly, males, being less educated, hypertension, diabetes, stroke, and a higher level of FBG and HCY than participants with low CSVD burden (*p* < 0.05). TTE parameters including E/A ratio, E/e′ ratio and LAD were significantly different between two groups (*p* < 0.05).

**TABLE 1:** Baseline characteristics of participants stratified by total CSVD score.

Characteristics	All (*N* = 3063)	Total CSVD scores = 2–4 (*n* = 255)	Total CSVD scores = 0–1 (*n* = 2808)	*p*
Age, years, mean ± SD	61.2 ± 6.7	67.1 ± 6.1	60.7 ± 6.5	< 0.001
Male, *n* (%)	1425 (46.5)	149 (58.4)	1276 (45.4)	< 0.001
Current smoker, *n* (%)	627 (20.5)	60 (23.5)	567 (20.2)	0.21
SBP, mmHg, median (IQR)	128.5 (118–139)	136 (125–148)	128 (117–139)	< 0.001
DBP, mmHg, median (IQR)	75 (69–81)	77 (72–83)	75 (69–81)	< 0.001
Education (≤ 6 years), *n* (%)	1361 (44.4)	146 (57.3)	1215 (43.3)	< 0.001
Medical history, *n* (%)				
Hypertension	1318 (43.0)	187 (73.3)	1131 (40.3)	< 0.001
Hyperlipidemia	614 (20.1)	44 (17.3)	570 (20.3)	0.24
Diabetes	662 (21.6)	78 (30.6)	584 (20.8)	< 0.001
Stroke	87 (2.8)	43 (16.9)	44 (1.6)	< 0.001
Cardiac disease	249 (8.1)	26 (10.2)	223 (7.9)	0.21
Laboratory results, mean ± SD				
TC, mmol/L	5.3 ± 1.0	5.2 ± 1.0	5.3 ± 1.0	0.15
LDL‐C, mmol/L	2.8 ± 0.8	2.7 ± 0.8	2.8 ± 0.8	0.23
FBG, mmol/L	6.0 ± 1.6	6.5 ± 2.8	5.9 ± 1.4	0.002
HCY	11.9 ± 6.2	14.2 ± 7.8	11.7 ± 6.0	< 0.001
TTE parameters				
LVEF, %, mean ± SD	67.8 ± 6.1	67.2 ± 5.9	67.9 ± 6.2	0.08
LVEF < 50, *n* (%)	20 (0.7)	3 (1.2)	17 (0.6)	0.50
LVFS, %, mean ± SD	38.0 ± 4.7	37.6 ± 4.6	38.0 ± 4.7	0.17
E/A ratio, median (IQR)	0.8 (0.7–1.1)	0.7 (0.6–0.8)	0.8 (0.7–1.1)	< 0.001
E/A ratio ≤ 0.8 or ≥ 2, *n* (%)	1473 (48.6)	184 (74.2)	1289 (46.3)	< 0.001
E/e′ ratio, median (IQR)	10.8 (8.8–13.2)	11.1 (9.0–14.2)	10.8 (8.8–13.1)	0.03
DT, ms, mean ± SD	183.0 ± 59.2	188.9 ± 70.3	182.5 ± 58.1	0.10
LAD, mm, mean ± SD	34.9 ± 4.7	36.1 ± 5.4	34.8 ± 4.7	< 0.001
LVEDD, mm, mean ± SD	47.8 ± 4.2	48.3 ± 4.4	47.8 ± 4.2	0.03

Abbreviations: BMI, body mass index; DBP, diastolic blood pressure; DT, deceleration time; FBG, fast blood glucose; HCY, homocysteine; IQR, interquartile range; LAD, left atrial diameter; LDL‐C, low density lipoprotein cholesterol; LVEDD, left ventricular end‐diastolic diameter; LVEF, left ventricular ejection fraction; LVFS, left ventricular fractional shortening; SBP, systolic blood pressure; SD, standard deviation; TC, total cholesterol; TTE, transthoracic echocardiogram.

### 3.2 The Association Between Cardiac Function and CSVD

In the multivariable logistic regression analysis, LVEF and LVFS, as continuous, were not associated with total CSVD score or CSVD imaging markers. After adjusting for confounders, E/A ratio showed a negative correlation with total CSVD score (cOR 0.89, 95% CI: 0.80–0.98, *p* = 0.01) (Table 2). For CSVD imaging markers, E/A ratio was correlated with lacunes (OR 0.63, 95% CI: 0.49–0.82, *p* < 0.001) and moderate to severe EPVS (OR 0.72, 95% CI: 0.60–0.87, *p* < 0.001). E/e′ ratio, DT, LAD, and LVEDD were not associated with total CSVD score or CSVD imaging markers. In addition, we further explored the association of TTE parameters with WMH or CMB stratified by the location of lesions. E/A ratio was correlated with PWMH (OR 0.89, 95% CI: 0.81–0.98, *p =* 0.02) and LAD was associated with lobar CMB (OR 0.78, 95% CI: 0.65–0.95, *p =* 0.01) (Table 3).

**TABLE 2:** Multivariable logistic regression analysis between cardiac function and total CSVD score or CSVD imaging markers.

Covariate	Total CSVD score		Lacune		Confluent WMH		CMB		Moderate to severe EPVS	
	cOR (95% CI)	*p*	OR (95% CI)	*p*	OR (95% CI)	*p*	OR (95% CI)	*p*	OR (95% CI)	*p*
LVEF, per SD	0.98 (0.91–1.07)	0.70	1.05 (0.89–1.25)	0.54	0.94 (0.85–1.043)	0.22	0.96 (0.86–1.09)	0.54	1.04 (0.91–1.18)	0.58
LVFS, per SD	0.97 (0.90–1.06)	0.51	1.04 (0.88–1.22)	0.68	0.93 (0.84–1.03)	0.16	0.96 (0.85–1.08)	0.50	1.03 (0.90–1.17)	0.70
E/A ratio, per SD	0.89 (0.80–0.98)	0.01	0.63 (0.49–0.82)	< 0.001	0.90 (0.79–1.02)	0.10	0.98 (0.85–1.12)	0.77	0.72 (0.60–0.87)	< 0.001
E/e′ ratio, per SD	0.98 (0.90–1.06)	0.59	1.06 (0.910–1.25)	0.50	0.97 (0.88–1.08)	0.62	1.07 (0.95–1.21)	0.25	0.96 (0.85–1.10)	0.57
DT, per SD	1.06 (0.98–1.15)	0.16	0.94 (0.80–1.10)	0.44	1.05 (0.95–1.16)	0.33	1.07 (0.95–1.21)	0.24	1.07 (0.95–1.21)	0.27
LAD, per SD	1.04 (0.96–1.13)	0.34	1.12 (0.95–1.31)	0.18	1.10 (0.99–1.22)	0.07	0.93 (0.83–1.05)	0.26	1.00 (0.88–1.13)	0.95
LVEDD, per SD	1.03 (0.94–1.13)	0.50	1.07 (0.89–1.28)	0.46	0.99 (0.89–1.12)	0.88	0.99 (0.87–1.13)	0.93	1.09 (0.95–1.25)	0.25

*Note:* Models are adjusted for age, sex, current smoker, systolic blood pressure, diastolic blood pressure, education level, history of hypertension, history of diabetes, history of hyperlipidemia, history of stroke, total cholesterol, low density lipoprotein cholesterol, fast blood glucose, homocysteine.

Abbreviations: CI, confidence interval; cOR, common odds ratio; DT, deceleration time; LAD, left atrial diameter; LVEDD, left ventricular end‐diastolic diameter; LVEF, left ventricular ejection fraction; LVFS, left ventricular fractional shortening; OR, odds ratio; SD, standard deviation.

**TABLE 3:** Multivariable logistic regression analysis between cardiac function and WMH, CMB stratified by the location of lesions.

Covariate	PWMH		DWMH		Lobar CMB		Deep/infratentorial CMB	
	OR (95% CI)	*p*	OR (95% CI)	*p*	OR (95% CI)	*p*	OR (95% CI)	*p*
LVEF, per SD	0.96 (0.88–1.04)	0.28	0.93 (0.84–1.03)	0.15	0.95 (0.80–1.14)	0.58	0.94 (0.78–1.12)	0.46
LVFS, per SD	0.95 (0.87–1.03)	0.19	0.91 (0.82–1.01)	0.08	0.95 (0.79–1.13)	0.54	0.93 (0.78–1.11)	0.43
E/A ratio, per SD	0.89 (0.81–0.98)	0.02	0.92 (0.80–1.04)	0.18	0.99 (0.81–1.21)	0.91	1.08 (0.90–1.31)	0.40
E/e′ ratio, per SD	1.03 (0.95–1.11)	0.55	0.98 (0.88–1.08)	0.63	0.95 (0.78–1.15)	0.58	1.16 (0.98–1.38)	0.09
DT, per SD	1.04 (0.89–1.22)	0.60	1.07 (0.97–1.19)	0.19	1.14 (0.95–1.36)	0.15	1.02 (0.86–1.22)	0.79
LAD, per SD	0.92 (0.85–1.00)	0.06	1.07 (0.97–1.19)	0.18	0.78 (0.65–0.95)	0.01	1.14 (0.96–1.36)	0.14
LVEDD, per SD	1.02 (0.94–1.11)	0.65	0.98 (0.87–1.09)	0.66	1.01 (0.83–1.22)	0.93	1.03 (0.85–1.26)	0.74

*Note:* Models are adjusted for age, sex, current smoker, systolic blood pressure, diastolic blood pressure, education level, history of hypertension, history of diabetes, history of hyperlipidemia, history of stroke, total cholesterol, low density lipoprotein cholesterol, fast blood glucose, homocysteine.

Abbreviations: CI, confidence interval; cOR, common odds ratio; DT, deceleration time; DWMH, deep WMH; LAD, left atrial diameter; LVEDD, left ventricular end‐diastolic diameter; LVEF, left ventricular ejection fraction; LVFS, left ventricular fractional shortening; OR, odds ratio; PWMH, periventricular WMH; SD, standard deviation.

**FIGURE 2:**
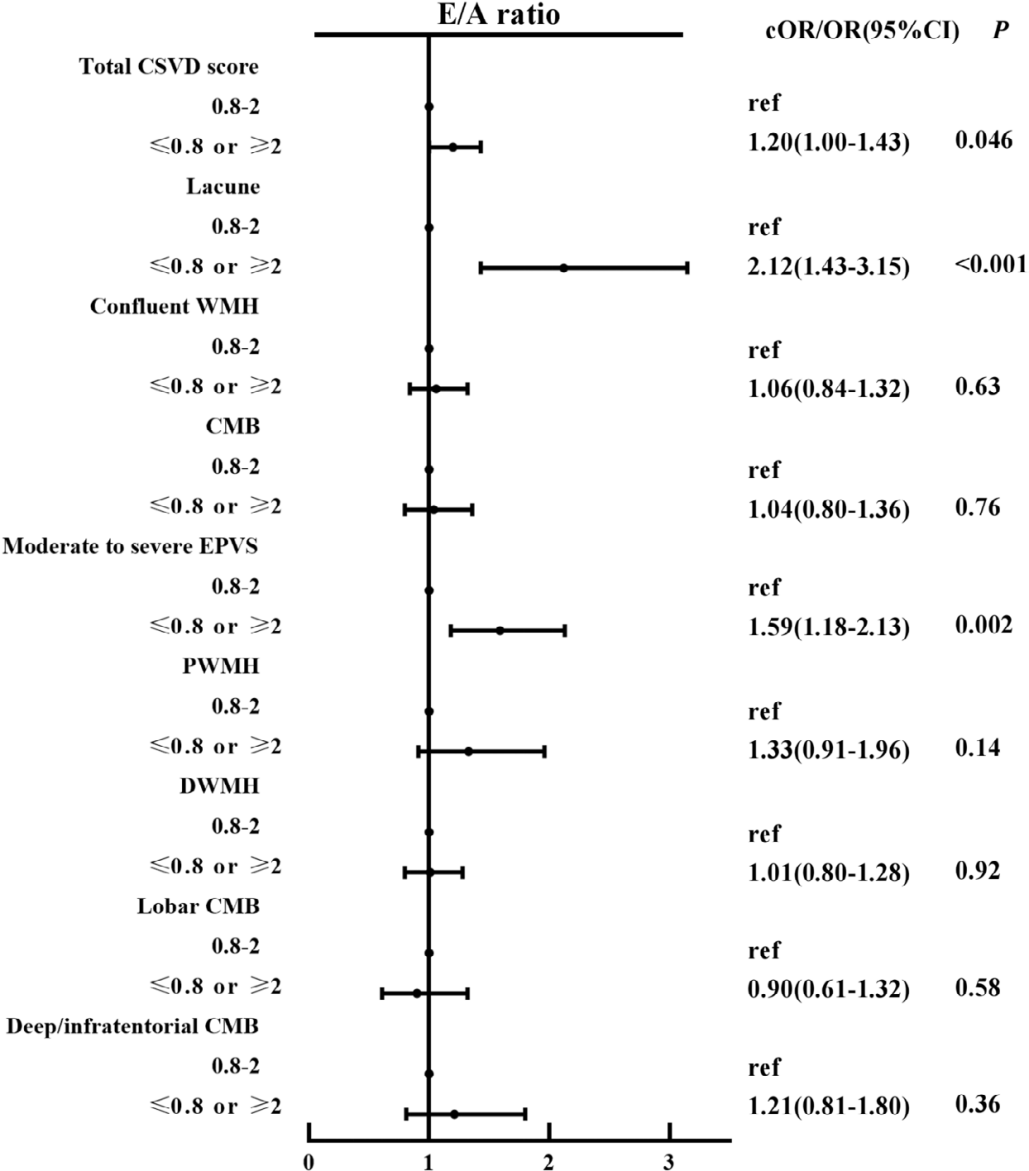
Forest plots for the association of E/A ratio with total CSVD score or CSVD imaging markers. Participants were divided into two groups according to E/A ratio (E/A ≤ 0.8 or ≥ 2 and 0.8 < E/A < 2). Taking the normal range (0.8 < E/A < 2) as the reference, multivariable logistic regression models were performed to explore the correlation between E/A ratio and total CSVD score or CSVD markers.

**FIGURE 3:**
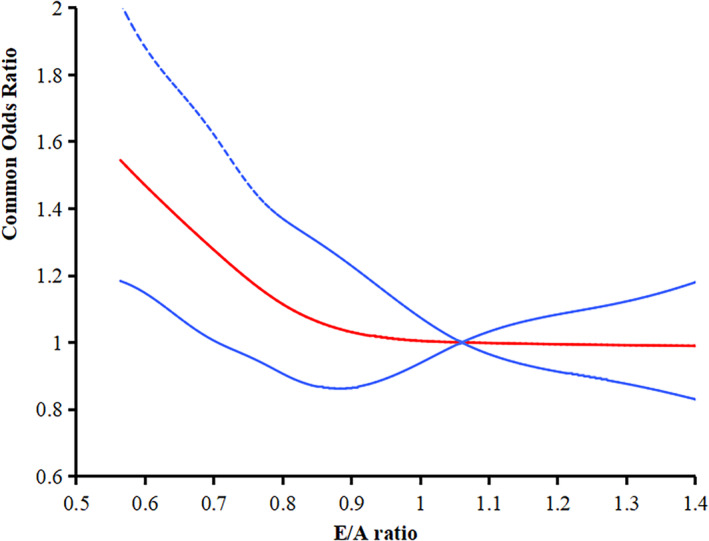
The relationship between E/A ratio and total CSVD score using a logistic regression model with restricted cubic spline. The solid red line indicates the adjusted common odds ratio and the blue lines the 95% confidence interval bands. Data were fitted using a logistic regression model with restricted cubic spline with five knots (the 5th, 25th, 50th, 75th, and 95th percentiles) for E/A ratio or E velocity, adjusting for potential covariates.

After adjustment for confounders, participants with E/A ≤ 0.8 or ≥ 2 had a higher total CSVD score than those with 0.8 < E/A < 2 (cOR 1.20, 95% CI: 1.00–1.43, *p =* 0.046) (Figure 2). For CSVD imaging markers, participants with E/A ≤ 0.8 or ≥ 2 had a higher risk of lacunes (OR 2.12, 95% CI: 1.43–3.15, *p* < 0.001) and moderate to severe EPVS (OR 1.59, 95% CI: 1.18–2.13, *p =* 0.002) as compared with those with 0.8 < E/A < 2, but not confluent WMH or CMB. Logistic regression analyses with restricted cubic spline further demonstrated that a lower E/A ratio were associated with a higher total CSVD score (Figure 3).

## 4 Discussion

In this study, our findings showed that LVEF and LVFS were not associated with total CSVD score or CSVD imaging markers. Furthermore, independent of vascular risk factors, participants with E/A ≤ 0.8 or ≥ 2 had a higher total CSVD score than those with 0.8 < E/A < 2. E/A ratio was correlated with nonhemorrhagic CSVD, including lacunes, moderate to severe EPVS and PWMH. Our results indicated that LV diastolic function was associated with CSVD. LV diastolic dysfunction might play a role in the pathophysiological mechanism of CSVD.

The pathogenesis of CSVD remains poorly illustrated. Chronic cerebral hypoperfusion is thought to be a potential mechanism [5]. LVEF has been used as an indicator for LV systolic function which is related to cerebral perfusion. Recent clinical studies have focused on the link between LV systolic function and CSVD, but its relationship remains controversial. Among acute ischemic stroke patients, LVEF values were negatively correlated with the severity of CSVD in a dose–response manner [9]. In a cross sectional analysis of UK Biobank, LVEF showed a negative association with WMH [10]. However, a retrospective study indicated that LVEF was not associated with CSVD among arteriosclerotic CSVD patients, although it could modify deep regional cerebral blood flow (CBF) [22]. In a community‐based cohort, LV GLS which can detect subclinical systolic dysfunction even when LVEF is normal was found to be independently associated with silent brain infarcts and WMH [11]. Likewise, our results showed no correlation between LVEF and total CSVD score or CSVD imaging markers, and the possible explanations might be as follows. First, the relationship between LV systolic function and CSVD may be more obvious in patients with acute ischemic stroke or chronic heart failure than the general population. Chronic hypoperfusion may be not the main mechanism of CSVD in community‐based population [23]. Second, LVEF values in the majority of participants (99.3%) enrolled in our study are in the normal range. Therefore, the alterations of LV systolic function in our study were mild. Longitudinal studies are needed to confirm the findings.

The relationship between LV diastolic function and CSVD has been relatively disregarded and rarely studied. Diastolic dysfunction is a condition that constitutes an insufficient filling of LV during the diastole due to impaired LV relaxation and increased LV stiffness [24, 25]. Evaluation of LV diastolic function is more challenging than systolic function [26]. E/A ratio and E/e′ ratio can be altered in diastolic dysfunction [25]. A previous study found that E/e' ratio and E/A ratio were significant predictors for CSVD in ischemic stroke patients [9]. Among arteriosclerotic CSVD patients, a study found that E/A ratio was negatively correlated with deep WMH, but no significant correlation existed between E/A ratio and deep regional CBF, indicating diastolic function may not play a role in deep regional perfusion [22]. E/e′ ratio was associated with the long‐term progression rate of WMH in population with preserved LV systolic function [12]. Our study also concluded that E/A ratio was associated with CSVD. The phenomenon may be explained by the following pathophysiology. First, vascular stiffness is mostly related to LV diastolic function. The link between diastolic function and CSVD indicated that vascular stiffness contributed to the development of CSVD [27, 28]. Second, small vessel disease is a multisystem disorder with a common pathophysiological basis that can affect both the heart and brain [29]. Endothelial dysfunction, inflammation and coagulation are the common pathways involved in microvascular dysfunction [30]. Last, it may be simple coincidence that some vascular risk factors are shared by patients with diastolic dysfunction and CSVD, and these vascular risk factors simultaneously progress to subclinical myocardial damage and the development of CSVD. In the future, further studies are needed to demonstrate the association between LV diastolic dysfunction and the long‐term progression of CSVD.

There were several limitations in the study. First. it is a cross sectional study lack of follow‐up data to validate the causal relationship between LV diastolic function and CSVD. In the future, we will evaluate the dynamics of the associations between the temporal changes of the TTE parameters and the possible progression of CSVD in a cohort of CSVD patients. Second, although we had a large sample with extensive measurements of CSVD and cardiac function, the participants enrolled in this study were from one city in China, which may have resulted in selection bias and inaccuracy to some degree. Third, the relationship between E/A ratio and LV diastolic dysfunction is complex. In our study, we considered E/A ratio ≤ 0.8 or E/A ratio ≥ 2 as LV diastolic dysfunction and the assessment of LV diastolic dysfunction was incomplete. Forth, considering that Fazekas scale is commonly used for semiquantitative assessment in clinical practice and WMH was based on Fazekas scale in the calculation of the total CSVD score, we adopted Fazekas scale to evaluate WMH, instead of quantifying WMH volume more accurately. Last, the unbalanced sample size between the high CSVD burden group and the low CSVD burden group might result in rare event bias, low predictive efficiency, and increased variance and reduced robustness in multivariable logistic regression analysis.

In conclusion, our study provides valuable support for an association between LV diastolic function and nonhemorrhagic CSVD. LV diastolic dysfunction assessed by TTE provide clues for the early warning of high CSVD burden. This study indicates novel insights about determining the pathophysiology of CSVD. The findings should be confirmed by large longitudinal studies.

## Conflicts of Interest

The authors declare no conflicts of interest.

## Supporting information


Table S1.

## Data Availability

The data that support the findings of this study are available from the corresponding author upon reasonable request.
