# Supplementary material for: Association of Left Ventricular Function With Cerebral Small Vessel Disease in a Community‐Based Population
Source: CNS Neurosci Ther. 2025 Feb 20;31(2):e70226. doi: 10.1111/cns.70226 (PMC11840704; doi:10.1111/cns.70226)
Supplement: Supplementary file 1 — Table S1. [file CNS-31-e70226-s001.docx]

| **Table S1.** Scanner parameters of brain MRI sequences | | | | |
| --- | --- | --- | --- | --- |
| Sequences | Orientation | Voxel /mm³ | Parameters | Scan time |
| T1W MPRAGE | 3D sagittal | 1.00×1.00×1.00 | TE=3.0ms, TR=6.7ms, TI=880ms, shot interval=2000ms, Flip angle=8° | 4:30 |
| SWI | 3D axial | 0.63×0.63×0.80 | first TE=7.2ms, echo spacing =6.2ms, 5echoes, Flip angle=17°, TR=37ms | 2:20 |
| FLAIR imaging | 2D axial | 0.53×0.53×6.50 | IE=110ms,TR=7000ms, TI=2300ms, SPIR fat suppression | 2:27 |
| T2W imaging | 2D axial | 0.51×0.51×6.50 | TE=105ms, TR=2500ms, SPIR fat suppression | 1:05 |
